# Living with and dying from advanced heart failure: understanding the needs of older patients at the end of life

**DOI:** 10.1186/s12877-015-0124-y

**Published:** 2015-10-15

**Authors:** Katharina Klindtworth, Peter Oster, Klaus Hager, Olaf Krause, Jutta Bleidorn, Nils Schneider

**Affiliations:** Institute for General Practice, Hannover Medical School, Carl-Neuberg-Str. 1, 30625 Hanover, Germany; AGAPLESION Bethanien Hospital, Geriatric Centre at the University, Heidelberg, Germany; Diakoniekrankenhaus Henriettenstiftung, Centre for Geriatrics, Hannover, Germany

**Keywords:** Older patients, Advanced heart failure, Palliative care, End of life, General practice, Needs, Patient perspective, Health care service, Advanced care planning

## Abstract

**Background:**

Heart failure (HF) is a life-limiting illness and patients with advanced heart failure often suffer from severe physical and psychosocial symptoms. Particularly in older patients, HF often occurs in conjunction with other chronic diseases, resulting in complex co-morbidity. This study aims to understand how old and very old patients with advanced HF perceive their disease and to identify their medical, psychosocial and information needs, focusing on the last phase of life.

**Methods:**

Qualitative longitudinal interview study with old and very old patients (≥70 years) with severe HF (NYHA III-IV). Interviews were conducted at three-month intervals over a period of up to 18 months and were analysed using qualitative methods in relation to Grounded Theory.

**Results:**

A total of 95 qualitative interviews with 25 patients were conducted and analysed. The following key categories were developed: (1a) dealing with advanced heart failure and ageing, (1b) dealing with end of life; (2a) perceptions regarding care, and (2b) interpersonal relations. Overall, our data show that older patients do not experience HF as a life-limiting disease. Functional restrictions and changed conditions leading to problems in daily life activities were often their prime concerns. The needs and priorities of older HF patients vary depending on their disease status and individual preferences. Pain resulting in reduced quality of life is an example of a major symptom requiring treatment. Many older HF patients lack sufficient knowledge about their condition and its prognosis, particularly concerning emergency situations and end of life issues, and many expressed a wish for open discussions. From the patients’ perspective, there is a need for improvement in interaction with health care professionals, and limits in treatment and medical care are not openly discussed.

**Conclusion:**

Old and very old patients with advanced HF often do not acknowledge the seriousness and severity of the disease. Their communication with physicians predominantly focuses on curative treatment. Therefore, aspects such as self-management of the disease, dealing with emergency situations and end-of-life issues should be addressed more prominently. An advanced care planning (ACP) programme for heart disease in older people could be an option to improve patient-centred care.

## Background

Much is known about heart failure, its treatment and medications. Guidelines facilitate targeted and standardised diagnoses and treatments. From a clinical point of view, heart failure often presents as a gradual functional decline with intermittent episodes of acute deterioration, some recovery, and a rather sudden and seemingly unexpected death [1, 2]. Particularly in older patients, heart failure often occurs in conjunction with other chronic disease, resulting in complex co-morbidity [3]. Heart failure affects approximately 2–5 % of adults aged 65–75 and >10 % of adults aged 80 and older [4, 5]. Patients with advanced cardiac failure often suffer from severe physical und psychosocial symptoms such as shortness of breath, pain, fatigue and fear as well as social isolation and restrictions regarding activities associated with daily living [3, 6–8]. As the proportion of older people is growing constantly, heart failure is becoming an ever-increasing public health problem [9]. Heart failure is a life-limiting illness for many patients. Half of all HF patients die within four years of diagnosis, and more than 50 % of those with severe heart failure die within one year [10]. Especially in old age, the likelihood of heart failure as the cause of death increases.

Palliative care is dedicated to patients with advanced life-threatening illnesses in order to improve their quality of life by addressing their psycho-social, physical and spiritual needs [11]. Better palliative care for older people is an international goal [11, 12]. Compared to cancer patients, patients with non-malignant diseases such as heart failure still have less access to palliative care services, even though an increasing numbers of such patients are in need of palliative care [6]. Studies show that palliative care plays an important role in relieving suffering and distress in patients and caregivers by providing optimal symptom management as well as psychological and spiritual support [7, 13]. End stage heart failure can be appropriately managed by using generic palliative care skills [14]. Moreover, palliative care helps in overcoming communication difficulties by addressing personal treatment goals and end of life issues such as advance directives and hospice care [7]. Previous studies focussed on a broad range of ages and did not explicitly deal with the needs and experiences of the old and very old^1^ [6, 15].

The aim of this study was therefore to understand how old and very old patients perceive advanced heart failure and to assess their medical, psychosocial and information needs at the end of life. In order to gain a deeper insight into patients’ perceptions and understanding of advanced chronic heart failure, we chose a qualitative longitudinal design.

## Method

### Study design

A qualitative longitudinal approach was chosen to better understand the patients’ dynamic illness experience and to determine how their physical, psychological, social and spiritual needs and use of health care services may vary over time. Other studies show that follow-up rounds of data collection allow time for participants to disclose information at their own pace and help to establish empathy and trust between patients and researchers [6].

### Patient recruitment

We recruited patients in two geriatric hospitals in the cities of Hannover and Heidelberg, which are located in northern and southern Germany, respectively. Both centres are academic teaching hospitals with a wide range of geriatric inpatient and outpatient services, including acute geriatrics and rehabilitation. Their overall treatment goals are the maintenance or recovery of good health, independence and, above all, quality of life for patients with age-related health problems caused by any geriatric illness, such as heart failure, stroke, fractures, or dementia. Parallel to acute medical treatment, a mobilisation and rehabilitation programme is usually started from the first day of hospitalisation. The patients are treated by experienced multidisciplinary teams in which physiotherapists, occupational therapists, speech therapists, massage therapists and balneotherapists work closely together with the nursing staff and physicians. After their stay, over 80 % of patients can return home.

Patients were recruited purposively over a period of 8 months (04-11/2010) until a sample size of 25 patients was reached [16]. Inclusion criteria were severe heart failure according to the New York Heart Association (NYHA) class III/IV, age ≥70 years and the ability to converse in German. There were two main reasons for choosing 70 years as the lower age cut-off. Firstly, the prevalence of heart failure increases considerably with age: population studies show that the overall prevalence rate of 8–16 cases/1000 increases to 40–60 cases/1000 among those aged >70 years [4]. Secondly, the clinical observation of the treating geriatricians in the recruitment hospitals indicated that end of life issues become more relevant in patients aged 70 years and older. Exclusion criteria were progressive cancer, moderate to severe dementia (Mini-Mental Exam score of <24) or frailty according to the CHSA Clinical Frailty Scale [17]. The “surprise” question—*“Would I be surprised if this patient died within the next 6 months?”—*helped senior geriatric physicians initially identify study participants during hospitalisation. This question has proved useful for identifying patients with a potential need for palliative care [18]. Initial information regarding the study was provided to potential participants verbally and via an information sheet by the treating senior physician. Of 29 patients approached after having been identified by a negative response to the surprise question, 25 agreed to participate in the study (9 in Hannover, 16 in Heidelberg). At this point, the interviewer approached the patients and provided further information regarding the study. After giving these individuals the opportunity to ask any outstanding questions and explaining that they were permitted to withdraw from the study at any time, participants were asked to provide written informed consent and agree to the recording of interviews. The study was approved by the local research ethics committee (Hannover Medical School, Nr. 5387, 25-08-2009).

### Data generation

Initial in-depth interviews were conducted shortly after patient recruitment in the hospital, and subsequent interviews took place at the patient’s home. If the patient desired, an informal carer (e.g. family member) was present. All interviews were conducted by the first author (KK). Comprehensive field notes were taken during the interview to gather data on non-verbal reactions and the course of the interview in order to facilitate contextualisation of the data. An interview postscript was written shortly after the interviews to record the context, atmosphere and the interviewer’s subjective impressions (e.g. feelings or odours) for each interview. A clinical assessment was performed once at the recruitment stage by the geriatric physician, which included the NYHA status and the frailty status, which was measured using the CHSA Clinical Frailty Scale [17]. Information about the involved professional carer (e.g. specialised physicians, nursing and social services) and informal carers (e.g. family members, neighbours) contextualised the qualitative findings.

The interview guide covered the patients’ experiences with heart failure, their main concerns at present (physical, psychological, social or spiritual), views on their care and treatment, and information about their condition and treatment. The guide was developed based on instruments previously used in other qualitative studies [6, 15, 19, 20]. The interview guide used for the sequential interviews included the same key topics as the one employed in the first interview, whilst focusing predominantly on perceived changes in the patient’s illness trajectory and life, and deepening individual issues identified in previous interviews.

**Fig. 1:**
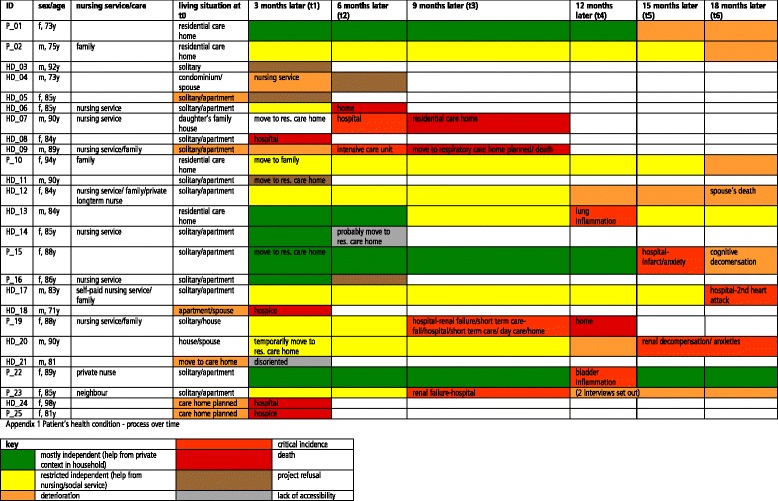
Changes in the patients’ health condition during the investigation

It was planned to conduct follow-up interviews at approximately three-month intervals, for up to seven interviews over a period of 18 months per patient (T0-T6). Of the 25 patients recruited, five refused to attend follow-up interviews, two were cognitively unable to respond, and eight died. At the end of the investigation period, a total of 95 interviews had been conducted with a mean length of 60 min (range 17–132 min); the attrition rate was 60 % (15 patients). Changes in the patients’ health condition during the investigation period are described in Fig. 1.

### Analysis

Interviews were audio-recorded and transcribed verbatim. All transcripts were checked with the audio file and thoroughly anonymised. The iterative analysis started after the first interviews. Researchers’ field notes and interview postscripts were used to enhance the interpretation. The qualitative analysis was performed using an inductive approach according to the principles of Grounded Theory [21]. Starting with a careful look at the transcripts, we openly coded relevant passages in the interviews with respect to the research questions using the qualitative data analysis software MAXQDA 10® [22]. First coding steps were conducted independently by two researchers and subsequently synthesised. The researchers’ field notes, interview postscripts and interview memos were used to enhance the joint interpretation. During the process, codes were subsumed alongside main categories into subcategories. Additionally, we conducted in-depth analysis of relevant passages to identify latent structures of meaning. Frequent team meetings (with experts in public health, nursing care, sociology, general medicine and palliative care) intensified reflection on the data.

## Results

25 patients (14 women/11 men) with a mean age of 85 years were included in the study [Table 1].

**Table 1:** Characteristics of the sample

Characteristics of the sample (*n* = 25)	
Sex female/male	14 (56 %)/11 (44 %)
Mean age (range)	85 years (71–98)
Marital status single/widowed	20 (80 %)
Living situation	
• Home	18 (72 %)
• Assisted living^a^/Nursing care home	7 (28 %)
Main care support	
• Family	8 (32 %)
• Spouse/partner	5 (20 %)
• Nursing service	9 (36 %)

^a^Assisted living = Institutions in Germany where persons live in their own apartment with optional care and household services

Our first impression when reading the field notes and interview material was that heart failure is not the prime concern of the interviewees. During the analysis, it became obvious that some older individuals labelled “cardiac failure patients” by their physicians did not perceive themselves as such. Further analysis of the material revealed two main themes, both of which were divided into various sub-categories: (A) Patient understanding of disease and prognosis and (B) Delivery of health care [Table 2].

**Table 2:** Categories

A. Patient understanding of disease and prognosis	B. Delivery of health care
1. Dealing with advanced heart failure and ageing	1. Perceptions regarding care
a. Perception of heart failure	a. Appropriateness of (medical) care
b. Adaption to changing conditions	
c. Appraisal of quality of life	b. Continuity of care
d. Information regarding the illness	2. Interpersonal relations
2. Dealing with the end of life	a. Interaction in the processes of care
a. Value and worthlessness in old age	
b. Preparation for death	b. Specific aspects in physician-patient interaction
	3. Meaning of family^a^

^a^This category is briefly introduced here and will be outlined in detail in the first author’s doctoral thesis, which will be published elsewhere

### A: Patient understanding of disease and prognosis

#### A1: Dealing with advanced heart failure and ageing

Patient statements revealed different levels of perspectives related to heart failure, ranging from descriptions of their illness (a) adaption to changing conditions (b), appraisal of their own quality of life (c), and their handling of information regarding their illness (d).

A1.a: Perception of heart failure

The patients described various experiences with symptoms of heart failure, in particular, limitations such as shortness of breath, dizziness, and restrictions in activities of daily living. Frequently, they did not perceive heart failure as a life-limiting, chronic disease with a long-term course, but rather as a disease with acute life-threatening events. The patients often believed their condition was a result of their old age and not a genuine illness. Medical definitions and personal experiences may diverge: Despite their specific diagnosis and treatment, the patients reported feeling well. Consequently, they may play down their symptoms and not take their medications. However, changes in the patients’ perceptions were sometimes observed when their condition worsened:

*Patient P1, T3 (73 y/o female): But my heart, that’s not really the problem. (…) That is (…) high blood pressure. Yes, I have that! Oh, yeah! You know, now … now I have it under control. You know, I have never taken any pills. (…) Yeah, and I watch out for my blood pressure. But if I say I take my tablets, that … I do not do that anymore.*

The loss of familiar concepts of self, such as body image and mental integrity, appear to constitute the main problem. Thus, the interviewees considered their mobility and participation in everyday life to be of greater importance than heart failure or any other disease itself.

*Patient HD20, T3 (90 y/o male): I’m feeling, shall we say, “old”. Yes. Well, the physical weakness can’t be overlooked so that takes precedence. And still, the heart pain is not so severe that I would say that my heart is the top priority. (…) I don’t feel like I have heart disease. Yes, I feel restricted in … movement, restricted in the range of activity, but compared to my heart, I would say that my knees aren’t as mobile. They stop me from walking or running properly, which is actually worse than the shortness of breath (…).*

This patient, a very old man who moved to a nursing home for a while to recover physically, had hoped to be able to live at home with his wife again. However, during the subsequent interview period (9 months), further health restrictions developed which, in the patient’s perception, were more important than the heart failure:

*Patient HD20, T5 (90 y/o male): Well, my kidney values … I have not paid any attention to them to date. The creatinine levels deteriorated rapidly. (…) And I’m really afraid of that. This I tell you frankly: I’m afraid, because I know what that means. That means weekly visits for, say, haemodialysis, which takes four to five hours, and to the doctor or for treatment … coming back here or wherever. (…) And that is so … it’s so … my, my … I would say my daily routine. But that is my main … my main problem. That is … you know, my kidney values are very bad, they are still working an average thirty percent. I don’t notice anything. Well, yes, I do notice something… um, my heart force levels are still at fifty percent. The big problem in my case is water retention throughout the body.*

A.1b: Adaption to changing conditions

Psycho-emotional strategies and practical assistance in daily life help the patients to adapt to physical and mental changes in their lives. Depending on individual attitudes in dealing with disease and age-related restrictions, these changes may be accepted or resisted. In some cases, physical and emotional boundaries were reluctantly adopted with time and led to a conscious approach to dealing with heart disease.

*Patient HD20, T0 (90 y/o male): [laughs] Yeah, I have to take it easy … take it easy, no longer pursue everything in my thoughts and desires, but I have to hold back. The heart somehow says: “Stop, don’t overdo it. You can’t want everything. But what you do take on, don’t do as much.” But that … that’s what I’m starting to see. But [laughs] not yet. Not yet.*

*I: Are you still in the process of doing so?*

*P: Yes, it’s hard enough cause when you are shown your own limits it’s sometimes painful.*

Another strategy used by the participants to come to terms more easily with their situation and to be able to better enjoy the present is to play down any discomfort. The disease duration can contribute to the perception that the illness and associated impairments are familiar and should not be given special attention any longer:

*Patient P15, T3 (93 y/o female): … Because that’s only at long intervals or because I’m used to it, that I get no … no air. This is perhaps through the many years that I already have, even from my young years, I was always a bit limited. Now I always think, “Relax and calm down, it will pass.”*

The patients reported various anxieties that may manifest as the illness progresses, fear of helplessness and uncertainty about future care arrangements. Moreover, the fluctuating course of heart failure with repeated acute incidents and accompanying diseases (multimorbidity) can provoke the fear of disease progression and deterioration of personal wellbeing.

*Patient P10, T1 (94 y/o female): And that [chest pain] … I don’t want to have that again. (…) No, not ever again, then the Lord God can take me back, but I couldn’t go through that again.*

In particular, the fear of losing mental function, which is expected with aging and the associated loss of independence, dominates in many cases:

*Patient P22, T6 (89 y/o female): Yes, if it’s still possible. Well, my greatest wish is and always was and will be that, in old age, I hopefully will not have the experience of noticing myself that I’m losing my mind. That is one thing that would be difficult for me. Although they always say that if you are demented, you don’t notice it anyway and so on, that is a … to me, that a rather horrific thought. (…) That is already a … a burden. I think not only for us old people, but also for .. for younger people, you know. Right, but it’s better if you push it to the back of your mind and say ((she laughs briefly)), it will not happen to me, no.*

III: Appraisal of quality of life

Older patients’ evaluation of their quality of life varies depending on the extent to which they retain their independence through activity and support. For those who largely appreciate their quality of life despite restricted physical wellbeing, aspects such as personality, a more positive attitude and social activity play an important role. This contrasts with older patients who complain of a significant loss of quality of life. Correlations were found between restriction of personal autonomy and the loss of enthusiasm for life. The loss of personal independence can lead to a feeling of absence of will and increasingly shifts the focus on basic care needs. This was observed in the case of a very old woman who was increasingly losing her will to live after moving into a nursing home, as identified at the fourth interview:

*Patient P15, T3 (93 y/o female): And it doesn’t really matter anymore if one comes to this. Then, eh, it is all the same to me now, and if one is completely run down, then I couldn’t care less, the main thing is that one has support and care.*

IV: Information regarding the illness

Overall, the interviewed cardiac failure patients had little knowledge about their condition and its limited prognosis. The subjective need for information varies according to disease state and individual preferences. The patients generally made little effort to actively acquire medical information outside the doctor’s office (at most, via low-threshold access sites, such as the media). Thus, the majority of their information was gained directly via doctor-patient communication.

Various motives regarding the wish to obtain information and knowledge (or not) related to how the patients dealt with their actual experience of the disease and were not inevitably related to the need to know medical facts. At T0, one patient joked about how she had refused information regarding her illness in the beginning due to fears; after experiencing a time of severe stages of the disease, she later seemed chastened and somewhat wiser, although she was still frightened of bad news.

*Patient P1, T6 (73 y/o female): And also in hospital, and as I know, I made a huge mistake: I have not always asked why, why, why. I didn’t want to know, no. (…) Yes, it’s my fault, I do not know how this could happen. Yes, if he [the doctor] tells me something, that I have such and such I’d get quite fuzzy-headed, then I’d get sick.*

Especially in emergency situations such as acute shortness of breath, the patients and their relatives reported that they had little idea regarding the course of action in such emergency situations. Subsequently, emergency responses are part of ‘everyday life’ for some patients.

#### A2: Dealing with the end of life

A2.a: Value and worthlessness in old age

Changes in the physical and living environment as well as the increasing loss of quality of life are decisive existential experiences for the interviewees that can lead to feelings of loneliness, sadness and indifference, and may increase a person’s wish to die. The feeling of boredom was also described: time can no longer be used in a fulfilling way because the necessary energy, mobility and means are lacking. In such cases, the patients longed for rather than rejected death and viewed life as having been lived. A main wish of the interviewed persons was to be able to carry out daily tasks without pain. Particularly when pain and suffering dominate everyday life, the desire to die increases over time and, after a certain point, some interviewees were considering shortening their life.

*Patient P23, T1 (85 y/o female): Because – I think, uh, life is, I don’t know, I do not know, it’s not worth living. Although I’m very attached to my grandchildren, a lot. (…) But apart from that, I always say one shouldn’t grow old.*

*Neighbour: Why? Eighty-six is not old?*

*P: Of course it is. It’s an old frigate.*

Nevertheless, the participants also expressed fears associated with the end of life. The wish for a peaceful, quick death without suffering was mentioned consistently.

*Patient P15, T1 (93 y/o female): Every day I think it may be the last, you know. (…) And I pray that I will fall asleep and never wake up again. (…) And I don’t wish anything else, just a pleasant parting, a fast one, yes, just falling asleep.*

Although most patients expressed a strong preference to die at home, specialist palliative care and facilities (e.g. hospices) were not discussed as an option to realise dying at home. At the same time, some of the interviewees did not reject hospitalisation if pain became unbearable.

A2.b: Preparation for death

Given the limited lifetime remaining, the respondents thought it particularly relevant that arrangements be made before they passed away. In all cases, the patients’ funeral was already planned and a will in place regarding their personal finances. Many patients said it was important to know that their family was provided for.

*Patient P1, T3 (73 y/o female): … But I was, well, a bit frightened … When you’re dead, then you’re gone. (…) Then I thought, “Oh my God! What will still be there and remain when you are dead and gone?” (…) You know, and now I say, everything is on order for my family, and that reassures me. Yes, that reassures me now.*

However, arrangements for the time before death, i.e. the process of dying and concomitant medical concerns, were often less definitively defined. While all interviewees did not want life-prolonging treatments, they had a range of strongly divergent ways to express their wishes: from written statements of intent (living wills and enduring powers of attorney) to oral delegation of decision-making power to family members or primary representatives. What is more, some made contradictory statements regarding the intention and implementation of advance directives.

*Patient HD17, T1 (83 y/o male): Well, if you, if we had strictly gone by the advance directive, I would not be sitting here today. Well, that is – for the life of me, I cannot imagine instructing someone: “You must absolutely do this”. And still one thinks, I think, the doctors, if there is still a possibility that I might pull through or not pull through, regardless of the advance directive. And if the doctors say there is .. there might be even a ray of hope, then I as a layman cannot say “No.” I see that, when you’re in the midst of pain, like the agonies I have had, then you say to yourself – I tell you in all honesty, I have often wished that [I might] just close my eyes and the suffering would come to an end. Because if you … It’s as someone had put a rope around your neck and is choking you, and doing that for three weeks. I couldn’t … I was gasping for air and could not breathe.*

Some patients, however, reject the living will completely because they see no need for this document or mistrust possible actions by physicians; thus, they are confident that their family will handle things without any written directions. Renewed inquiry during the sequential interview sessions suggested that this attitude did not change over time. For example, the following patient kept his strong confidence in the decision power of this family, which did not waver from T1 to T6:

*Patient P2, T1 (75 y/o male) You know, my family knows how I think and what I want if it gets to the point that, say, artificial feeding or something like that is required, they would absolutely decline that. That’s what I’ve said. And since I don’t make a big effort, I don’t write anything, afterwards they [KK: physicians] go from the beginning, don’t do that, don’t do this, let it be. My family knows exactly how I think and that’s the way it is.*

*T6 I: … An advance directive about life-prolonging treatment, whether you want that or not?*

*P: No, I do not want that. … But my children, they all know that I do not want that.*

*I: Have you talked to your doctor about it?*

*P: No, I haven’t, I don’t talk about stuff like that with my doctor! If I did, then I’d do it in the hospital. If I were admitted to the hospital now, then I would say, “Listen up, so and so, if something is wrong now, do not hook me up to tons of machines and equipment! I say, when it’s over, it’s over!”*

### B: Delivery of health care

#### B1: Perceptions regarding care

B1.a: Appropriateness of (medical) care

The interviewed patients’ assessment of the quality of medical and nursing care varied depending on whether they thought the treatment was appropriate, necessary and met their needs. If decisions about medical treatment, prescriptions and home visits made by the professionals do not meet a patient’s expectations, they may be perceived as inadequate or ‘wrong’. Treatment of pain was particularly highlighted: The fact that patients often perceive pain as an expression of complete suffering not attributable to any particular (organic) cause leads to a type of indifference that makes any medical help inconceivable. The following quotation (from a patient’s third interview session) clearly illustrates this perception of ubiquitous suffering, which the patient repeatedly mentioned in every interview.

*Patient HD12, T2 (84 y/o male): “I only want to feel better. But it seems it’s not to be, nothing good. Being ill, there’s nothing wanted, nothing needed, it’s always the same. (…) I don’t need anything, I can be quite alone. Pain everywhere (…)*

As revealed in the interviews, it seems that heart failure patients’ care needs vary during the course of illness depending on their current state of illness and individual requirements. In their individual preferences, the need for emergency intervention may be weighed against the wish for participatory decision-making. Quick action without the patient’s express permission may be acceptable in acute situations, while the need for participatory decision-making may prevail in the context of stable and consistent care. Thus, patients prioritize individual support preferences and negotiate specific treatment interventions depending on the situation.

*Patient HD06, T1 (85 y/o female): So you know, if a patient is really ill, yes, it’s good when you have someone who looks after you. I mean, I do not want too much care, it would be too much responsibility for me, you know. But if you’re not feeling well, it’s good to have someone.*

B1.b: Continuity of care

Transition situations, i.e. from hospital to home, often reveal gaps in the provision of care. Although all patients interviewed preferred to be cared for at home, joint discussions between doctor and patient regarding medical and therapeutic treatment options in ambulatory settings did not take place. One patient perceived recurrent visits to a day unit (in addition to visits by nursing and medical assistants) as stressful:

*Patient P22, T3 (89 y/o female): … After the [hospital] treatment, the professor indicated the only option was the day unit for this and that. And I told him I don’t want that, I don’t want to come daily. I’ve just come back from hospital.*

#### B2: Interpersonal relationships

B2.a: Interaction in the processes of care

In situations requiring increased nursing and medical care, the patients encountered different needs regarding both the intensity and frequency of interaction. Patients described situations that reveal a lack of attention and empathy at the professional and informal level. Increased care needs and concomitant dependencies can cause feelings of helplessness. The affected patients stated that they endure these situations because they cannot defend themselves and/or fear disadvantages in subsequent care if they complain.

*Patient P1, T1 (73 y/o female): … It was my first time in the hospital. Some of the nurses there were rather unpleasant. Because, you know, I couldn’t move, not at all. And if I rang, there was a long delay; I would lie there in pain on my back or askew. They also treated me so impersonally, and didn’t ask what the problem is or if it’s better. “Sorry, we don’t have time now.” Because of this, I swore to myself that I wouldn’t get so ill again that I would need to be in a hospital (…) And then I got a little better. And then they started talking to me … when I didn’t really need them anymore.*

B2.b: Specific aspects in physician-patient interaction

From the older HF patients’ perspective, communication with professional caregivers sometimes lacks authenticity. This was consistently reflected in statements that they did not feel recognised, heard or taken seriously. For example, when the patients reported symptoms such as shortness of breath or pain, they often got vague responses from their doctors, who put it down to old age or weather.

*Patient HD17, T4 (83 y/o male): And three days before I collapsed with a heart attack, I noticed something [was wrong], and then I went to the doctor, but she was not there (…) And her husband was also a doctor, so I went to him. “Oh my,” he says. I said, “Doctor, I’m not feeling well,” and so on. “Well, you have to consider your age, and also the weather.” Later I collapsed. He did not examine me, did not take my pulse or blood pressure; he didn’t do anything. He used to be a mason but re-trained. (…) But I think he’s still a bricklayer.*

This communication habit by professionals may not only lead to unanswered questions and dissatisfaction, but may also give older patients the impression that treatment options exist but are not being offered to them.

According to the respondents, it seems that the limits and possibilities of medical care and therapy are not being clearly addressed by health professionals. One of various problems in doctor-patient communication revealed in the interviews seems to be related to the doctors’ hesitation to articulate uncertainties. Many patients, on the other hand, would like to be informed and not be left in limbo when it comes to diagnosis or treatment.

*Patient HD 20, T0 (90 y/o male): Perhaps to more clearly emphasize the risks, the risks of the disease … the risks and dangers. Because he [KK: the GP] he had us there … Here and there, there in the reports, he said something about heart failure without saying what it actually meant. Or he mentioned, um, “palpitations”. Those are two examples that spontaneously come to mind now. Then, I would have liked for him to have put it to me more clearly, and that he had said, you have this and that and need to watch out for this.*

#### B3: Meaning of family

Informal caregivers, who are mostly family members, have a key role in health care support between patients and health professionals. They act and react on different levels: as an observer of professional health care (situations), as a link between the parties, and as actively involved carers while, at the same time, they are affected themselves. Our findings regarding the meaning of family were presented in a differentiated manner elaborating on diverse types of relationships (monad, dyad, and triad). These findings will be published elsewhere (please refer to Table 2).

## Discussion

The results of this study show that old and very old patients with advanced heart failure often do not perceive heart failure as a life-limiting disease. Restrictions of daily life weighed much more predominantly on their minds, irrespective of whether due to heart failure or other comorbidities. Pain resulting in reduced quality of life is an example of a major symptom requiring treatment in this population. The needs and priorities of older heart failure patients are diverse and vary according to the status of illness and individual preferences. Many older patients with cardiac failure lack sufficient knowledge of their disease, its prognosis and possible treatment options. Especially issues regarding the handling of emergency situations and end of life care planning raise questions, and many patients expressed a desire for open discussions of these issues. However, in this regard, interaction with health care professionals is unsatisfactory in the patients’ opinion. Many of the patients believe that their physicians do not openly communicate the limits of treatment and medical care to them.

Other studies of chronically ill patients confirm our findings that these patients’ primary concern is not the disease itself, but the disruption to their life [6, 23]. Contrary to the experiences of younger people with severe heart disease, the physical and social decline associated with co-morbidities, progressive losses and social isolation become the more prominent issues with increasing age [24]. However, old and very old heart failure patients show different concerns and needs during the course of illness with regard to both medical treatment/care and information/communication needs. This is consistent with the findings of Murray et al. [6, 15].

Overall, current studies indicate that many cardiac failure patients have a poor understanding of the disease and its prognosis [6, 25]. In several cases, the patients’ questions remain unanswered or they get information that they do not properly understand (25). One problem might be that some doctors insufficiently inform their patients that advanced heart failure is a serious and fatal disease. Perhaps due to a lack of confidence, barriers exist that hinder physicians from discussing a poor prognosis and end of life issues with their patients—a task that also requires a high level of communicative competence [26–29]. These aspects should be addressed in educational interventions designed to improve communication skills, particularly among primary care professionals [25]. Another problem may be a lack of sufficient discussion between patient and family physician on how to monitor and responsibly manage disease-related changes and exacerbations in daily life. An emergency plan drawn up by the patient in collaboration with the attending physician that specifies the desired place of death and end of life treatment preferences could fill this gap and avoid unnecessary emergency responses and hospital admissions – but many patients reject this approach [30, 31].

On the other hand, different patients may prefer different levels of information [32]. In principle, this should be respected. However, a patient’s refusal of information may be a strategy to protect himself or herself from the burden of knowledge. Some older patients play down discomfort as a strategy to facilitate their lives, deny disease-related damages and maintain social participation, regardless of the physical limitations imposed by heart disease [23]. Nevertheless, the risk of such denial strategies is that important complaints and concerns may not be identified and adequately addressed by the doctor, and possibilities for appropriate self-management may be neglected. In such a case, joint decisions are hardly possible and, particularly in emergency situations, decision-making is delegated to third-party physicians [33]. A collaborative relationship between the older patient and the patient’s main doctor (usually the family physician) enables participatory decision-making and sustains the vulnerable patient’s autonomy and dignity [34].

Similarly to other statements of intent, there are significant uncertainties concerning the effects of a written document regulating end of life issues. Firstly, it is hard for older patients to make decisions about treatment in disease-related crises. Secondly, the implications of fixed prior decisions are difficult to estimate, so it might be best to leave these decisions to the discretion of the patient’s doctors or relatives [33].

From a medical perspective, older patients with heart failure suffer from known problems, such as shortness of breath or weakness. However, older individuals may have very different perceptions and assessments of these objective and similar symptoms. Our analysis revealed that the patients’ perceptions, attitudes and preferences were not primarily determined by the stage of illness. In fact, the observed differences seem to be grounded in individual habits and attitudes acquired throughout life. This appears to result in personal priority settings, which may differ from those of the attending physician and other professionals involved in the patient’s care. Thus, it may be prudent for the involved (family) physicians to regularly assess the patient’s physical condition, expectations and needs as the basis of a structured discussion including information, medical treatment options and treatment limitations in order to set priorities in the participatory process. This could be part of a regular geriatric assessment conducted by the patient’s general practitioner (GP).

Thus, there is a need for anticipatory care planning including information and self-management strategies for daily life as well as emergencies involving both older patients and their carers [15]. The recurrent geriatric assessment should address questions regarding pain management and individual end of life issues. In this context, communication training of physicians dealing with older people is crucial and should be take into account the diverging needs and the individual. Uncertainties and limitations of treatment must be addressed in order to avoid inappropriate health care [35].

A disease-specific advance care planning (ACP) programme could help to facilitate discussions about end of life care preferences between patients, families and caregivers [36]. ACP aims to realise a patient’s autonomy and self-determination in medical decision-making at the end of life. An ACP programme tailored to patients with heart failure has the potential to improve patient-centred care while identifying the patient’s values, goals and preferences regarding treatment [37]. In principle, ACP could be performed by different professionals such as GPs, community nurses, social workers, nurses in care homes and hospital teams [38]. The implementation of an ACP programme depends on local health care structures, responsibilities and cooperation.

### Strengths and limitations of the study and method

Our study has a consistent and in-depth focus on the patients’ perspective. A major strength is the inclusion of very old patients (average 85 years). It is in the nature of qualitative studies to have a small number of participants. Therefore, the findings cannot be generalised, but they provide specific in-depth insights into the perception and personal experience of old and very old patients with severe heart failure. The patients were only recruited in an inpatient setting at two geriatric hospitals and not in general practice or other outpatient settings. Furthermore, the tendency towards socially desirable statements must be considered. To reduce social bias, the interviewer was not involved in the delivery of health care for the patients and was not employed by either of the geriatric hospitals.

The longitudinal design enabled the continuity of contact with patients, which helped to establish growing confidence and trust. This qualitative access allowed us to gain broad information on sensitive and personal issues, which greatly enriched the informative value of the generated data. In addition, the personal contact helped to prevent attrition. On the other hand, the objective to explore how the needs of old and very old people change throughout their illness trajectory could only be achieved to a limited degree. The interviewees’ perception and expression of needs appeared to be determined by the individual habits and attitudes they had adopted throughout life and, thus, remained stable and unchanged during the study phase.

## Conclusion

Old and very old patients with advanced heart failure often do not acknowledge the seriousness and severity of the disease. Their communication with physicians predominantly focuses on curative treatment. Therefore, aspects such as self-management of the disease, dealing with emergency situations and end-of-life issues should be addressed more prominently. An advanced care planning (ACP) programme for heart disease in older people could be an option to improve patient-centred care.

What this study adds:

1. Most patients with advanced heart failure do not perceive their condition as a life-limiting disease.
2. Many of these patients lack sufficient knowledge of their disease and its prognosis and do not have a plan for emergency situations.
3. Symptoms of heart disease are related to age and comorbidities.
4. These patients commonly play down and hide discomfort when communicating with physicians.
5. Thus, there is definitive need for special advanced care planning (ACP) programs for heart disease in the old and very old.

## Abbreviations

HF: Heart failure
ACP: Advanced care planning
NYHA: New York Heart Association
CHSA: Canadian Study of Health and Aging
MAXQDA®: Product name of a qualitative data analysis software

## Footnotes

1 Very old was defined as ≥ 80 years in this study.
